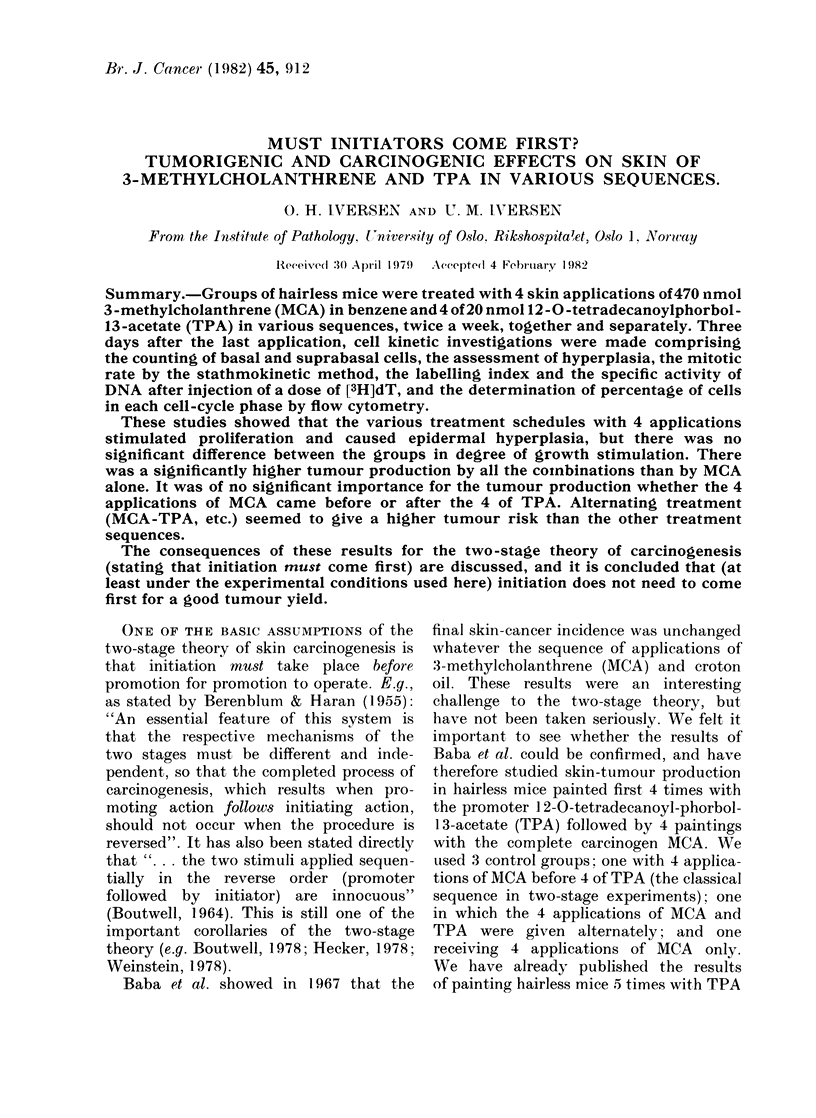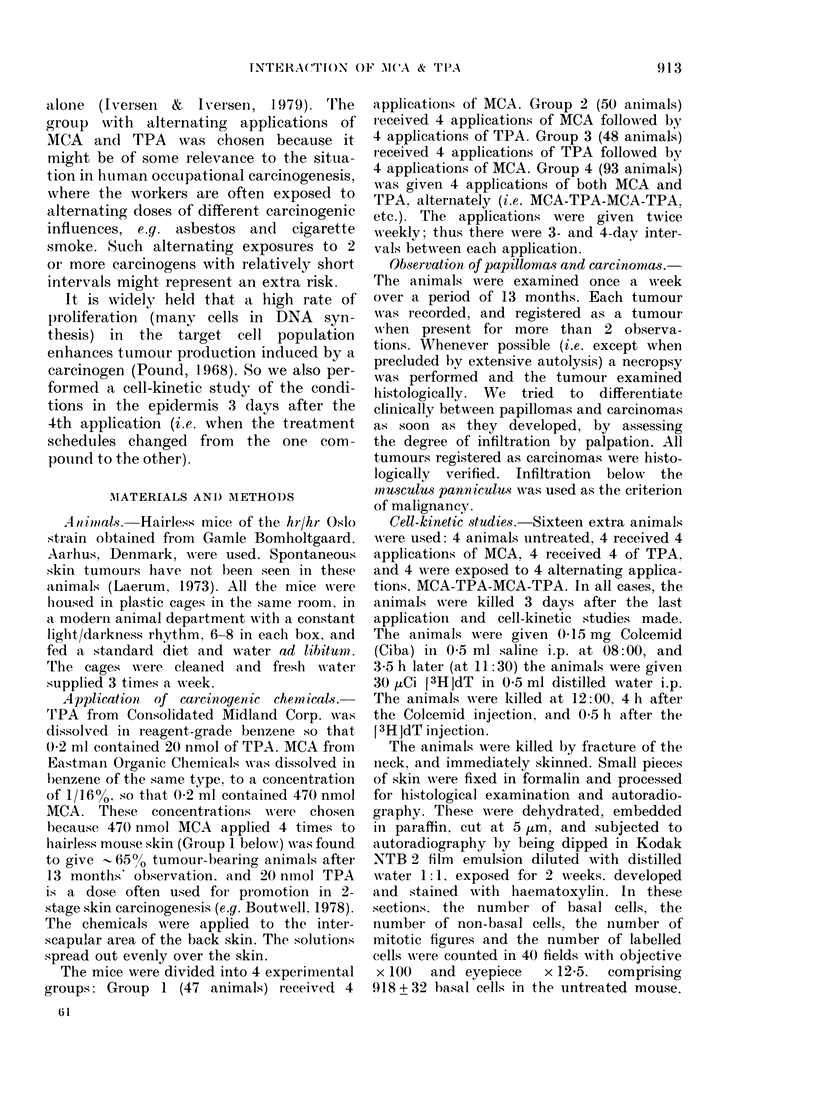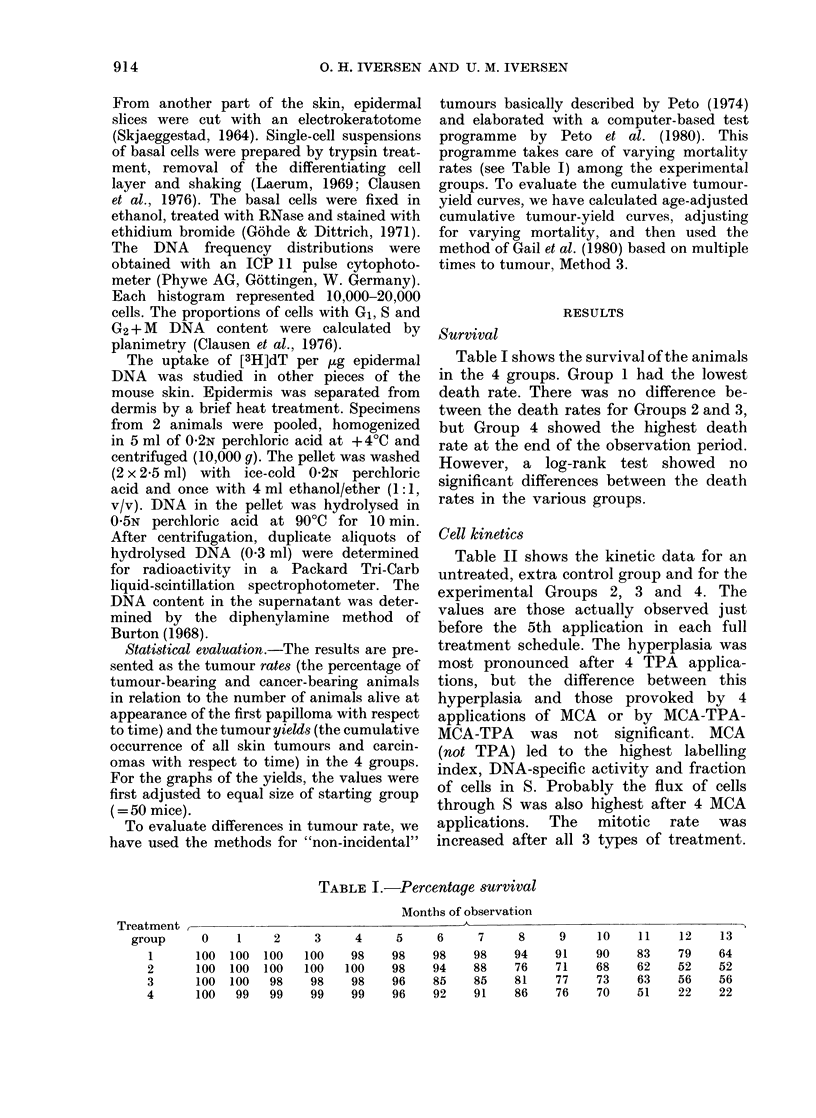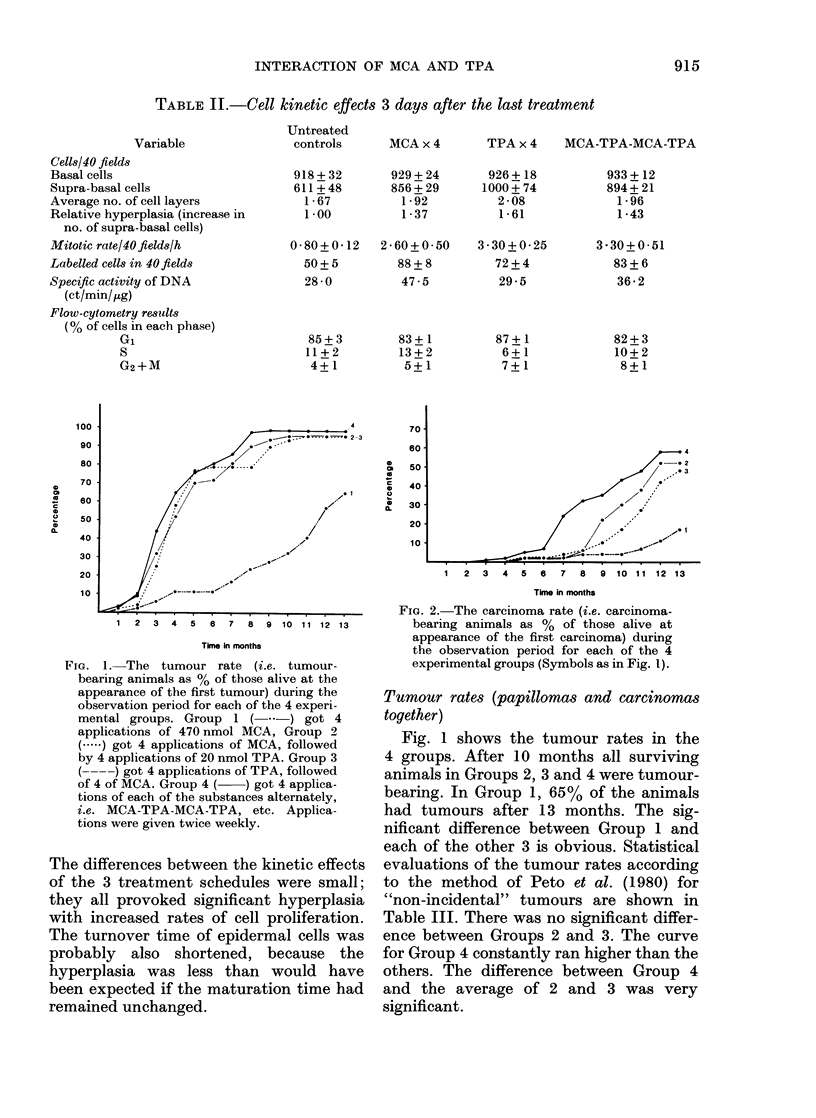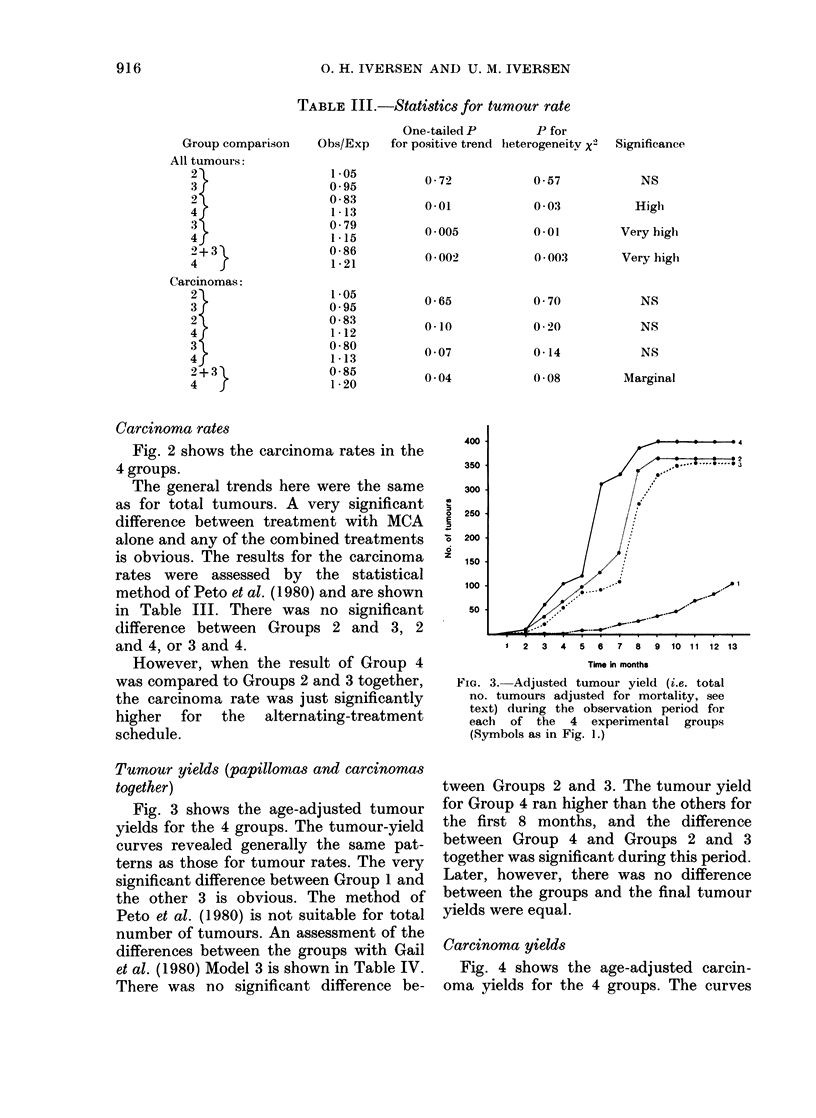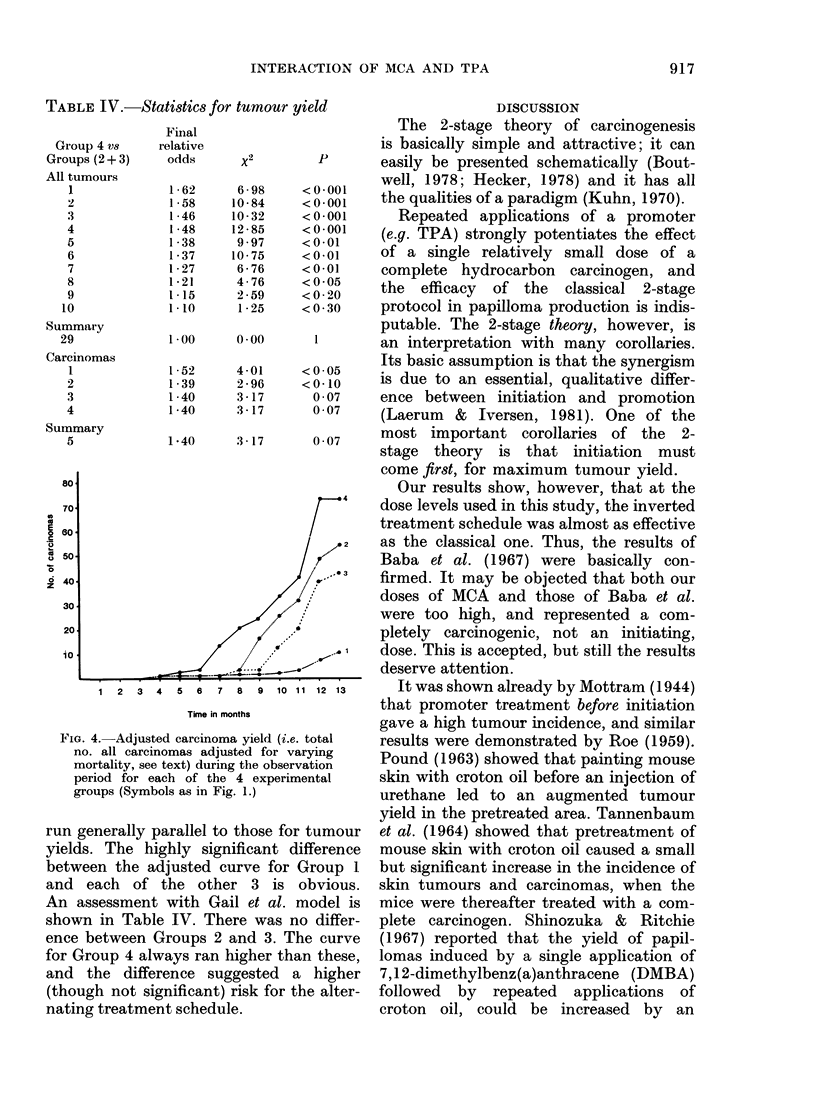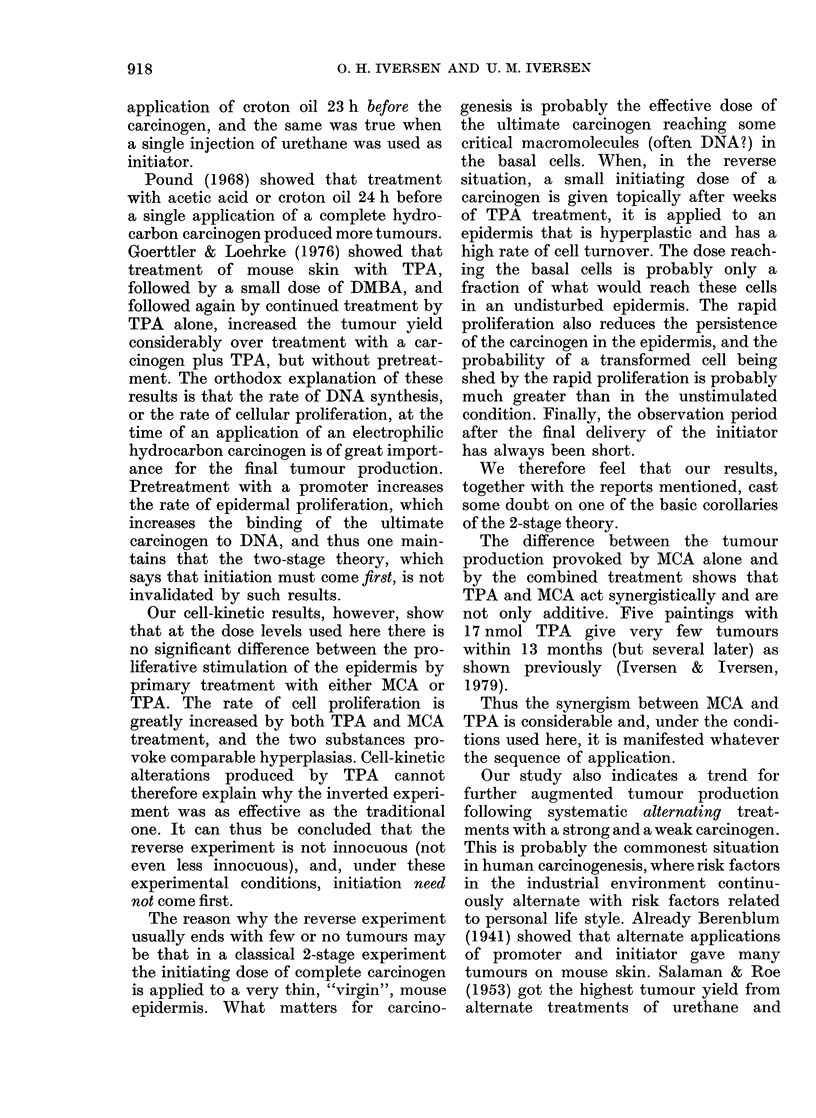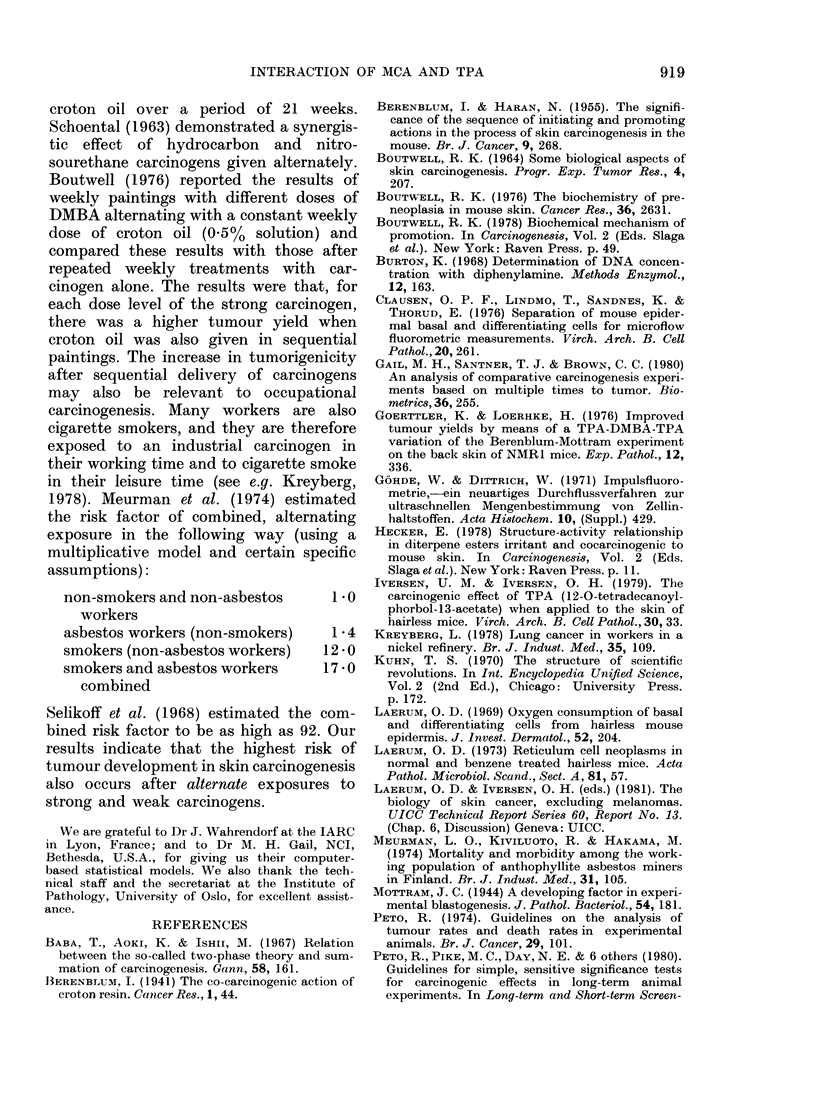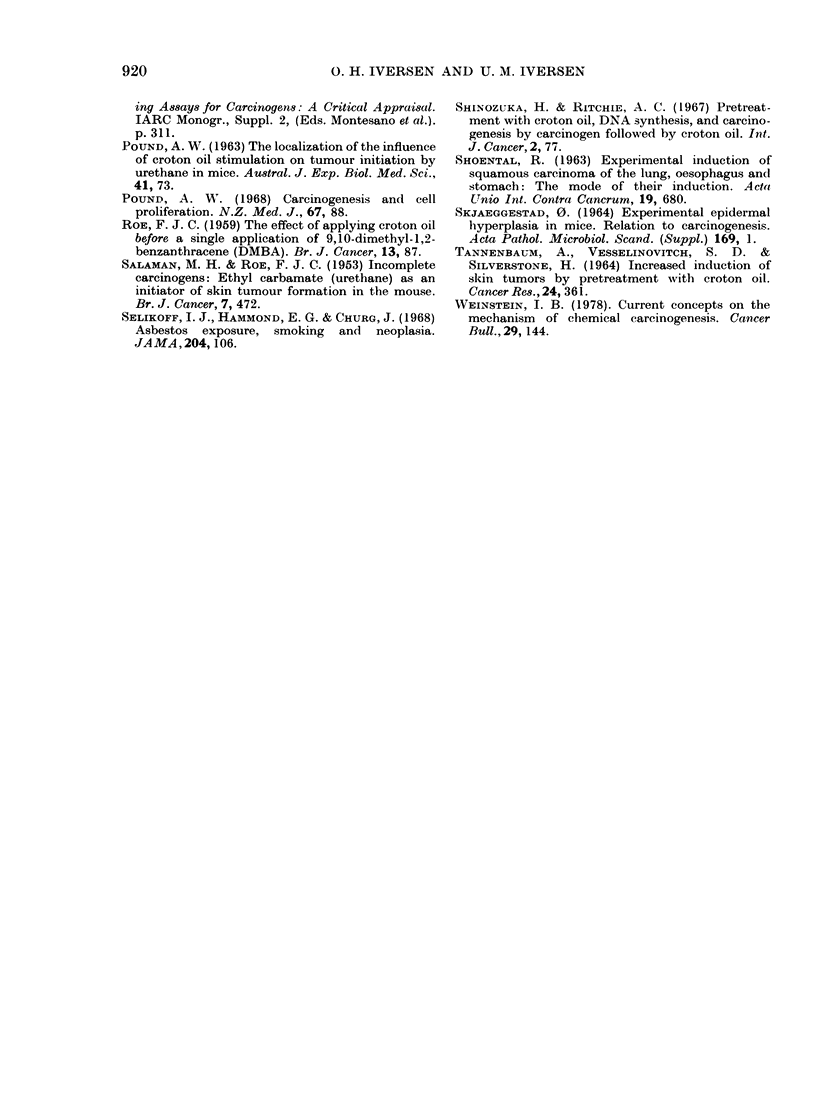# Must initiators come first? Tumorigenic and carcinogenic effects on skin of 3-methylcholanthrene and TPA in various sequences.

**DOI:** 10.1038/bjc.1982.144

**Published:** 1982-06

**Authors:** O. H. Iversen, U. M. Iversen

## Abstract

Groups of hairless mice were treated with 4 skin applications of 470 nmol 3-methylcholanthrene (MCA) in benzene and 4 of 20 nmol 12-O-tetradecanoylphorbol-13-acetate (TPA) in various sequences, twice a week, together and separately. Three days after the last application, cell kinetic investigations were made comprising the counting of basal and suprabasal cells, the assessment of hyperplasia, the mitotic rate by the stathmokinetic method, the labelling index and the specific activity of DNA after injection of a dose of [3H]dT, and the determination of percentage of cells in each cell-cycle phase by flow cytometry. These studies showed that various treatment schedules with 4 applications stimulated proliferation and caused epidermal hyperplasia, but there was no significant difference between the groups in degree of growth stimulation. There was a significantly higher tumour production by all the combinations than by MCA alone. It was of no significant importance for the tumour production whether the 4 applications of MCA came before or after the 4 of TPA. Alternating treatment (MCA-TPA, etc.) seemed to give a higher tumour risk than the other treatment sequences. The consequences of these results for the two-stage theory of carcinogenesis (stating that initiation must come first) are discussed, and it is concluded that (at least under the experimental conditions used here) initiation does not need to come first for a good tumour yield.


Br. J. Cancer (1982) 45, 912

### MUST INITIATORS COME FIRST? TUMORIGENIC AND CARCINOGENIC EFFECTS ON SKIN OF 3-METHYLCHOLANTHRENE AND TPA IN VARIOUS SEQUENCES.

O. H. IVERSEN AND U. M. IVERSEN

*From the Institute of Pathology, University of Oslo, Rikshospitalet, Oslo 1, Norway*

Received 30 April 1979 Accepted 4 February 1982

**Summary.—Groups of hairless mice were treated with 4 skin applications of 470 nmol 3-methylcholanthrene (MCA) in benzene and 4 of 20 nmol 12-O-tetradecanoylphorbol-13-acetate (TPA) in various sequences, twice a week, together and separately. Three days after the last application, cell kinetic investigations were made comprising the counting of basal and suprabasal cells, the assessment of hyperplasia, the mitotic rate by the stathmokinetic method, the labelling index and the specific activity of DNA after injection of a dose of [$^3$H]dT, and the determination of percentage of cells in each cell-cycle phase by flow cytometry.**

**These studies showed that the various treatment schedules with 4 applications stimulated proliferation and caused epidermal hyperplasia, but there was no significant difference between the groups in degree of growth stimulation. There was a significantly higher tumour production by all the combinations than by MCA alone. It was of no significant importance for the tumour production whether the 4 applications of MCA came before or after the 4 of TPA. Alternating treatment (MCA-TPA, etc.) seemed to give a higher tumour risk than the other treatment sequences.**

**The consequences of these results for the two-stage theory of carcinogenesis (stating that initiation *must* come first) are discussed, and it is concluded that (at least under the experimental conditions used here) initiation does not need to come first for a good tumour yield.**

ONE OF THE BASIC ASSUMPTIONS of the two-stage theory of skin carcinogenesis is that initiation *must* take place *before* promotion for promotion to operate. *E.g.*, as stated by Berenblum & Haran (1955): "An essential feature of this system is that the respective mechanisms of the two stages must be different and independent, so that the completed process of carcinogenesis, which results when promoting action *follows* initiating action, should not occur when the procedure is reversed". It has also been stated directly that ". . . the two stimuli applied sequentially in the reverse order (promoter followed by initiator) are innocuous" (Boutwell, 1964). This is still one of the important corollaries of the two-stage theory (*e.g.* Boutwell, 1978; Hecker, 1978; Weinstein, 1978).

Baba *et al.* showed in 1967 that the final skin-cancer incidence was unchanged whatever the sequence of applications of 3-methylcholanthrene (MCA) and croton oil. These results were an interesting challenge to the two-stage theory, but have not been taken seriously. We felt it important to see whether the results of Baba *et al.* could be confirmed, and have therefore studied skin-tumour production in hairless mice painted first 4 times with the promoter 12-O-tetradecanoyl-phorbol-13-acetate (TPA) followed by 4 paintings with the complete carcinogen MCA. We used 3 control groups; one with 4 applications of MCA before 4 of TPA (the classical sequence in two-stage experiments); one in which the 4 applications of MCA and TPA were given alternately; and one receiving 4 applications of MCA only. We have already published the results of painting hairless mice 5 times with TPA

alone (Iversen & Iversen, 1979). The group with alternating applications of MCA and TPA was chosen because it might be of some relevance to the situation in human occupational carcinogenesis, where the workers are often exposed to alternating doses of different carcinogenic influences, *e.g.* asbestos and cigarette smoke. Such alternating exposures to 2 or more carcinogens with relatively short intervals might represent an extra risk.

It is widely held that a high rate of proliferation (many cells in DNA synthesis) in the target cell population enhances tumour production induced by a carcinogen (Pound, 1968). So we also performed a cell-kinetic study of the conditions in the epidermis 3 days after the 4th application (*i.e.* when the treatment schedules changed from the one compound to the other).

### MATERIALS AND METHODS

*Animals*.—Hairless mice of the *hr/hr* Oslo strain obtained from Gamle Bomholtgaard, Aarhus, Denmark, were used. Spontaneous skin tumours have not been seen in these animals (Laerum, 1973). All the mice were housed in plastic cages in the same room, in a modern animal department with a constant light/darkness rhythm, 6–8 in each box, and fed a standard diet and water *ad libitum*. The cages were cleaned and fresh water supplied 3 times a week.

*Application of carcinogenic chemicals*.—TPA from Consolidated Midland Corp. was dissolved in reagent-grade benzene so that 0·2 ml contained 20 nmol of TPA. MCA from Eastman Organic Chemicals was dissolved in benzene of the same type, to a concentration of 1/16%, so that 0·2 ml contained 470 nmol MCA. These concentrations were chosen because 470 nmol MCA applied 4 times to hairless mouse skin (Group 1 below) was found to give $\sim$65% tumour-bearing animals after 13 months' observation, and 20 nmol TPA is a dose often used for promotion in 2-stage skin carcinogenesis (*e.g.* Boutwell, 1978). The chemicals were applied to the interscapular area of the back skin. The solutions spread out evenly over the skin.

The mice were divided into 4 experimental groups: Group 1 (47 animals) received 4 applications of MCA. Group 2 (50 animals) received 4 applications of MCA followed by 4 applications of TPA. Group 3 (48 animals) received 4 applications of TPA followed by 4 applications of MCA. Group 4 (93 animals) was given 4 applications of both MCA and TPA, alternately (*i.e.* MCA-TPA-MCA-TPA, etc.). The applications were given twice weekly; thus there were 3- and 4-day intervals between each application.

*Observation of papillomas and carcinomas*.—The animals were examined once a week over a period of 13 months. Each tumour was recorded, and registered as a tumour when present for more than 2 observations. Whenever possible (*i.e.* except when precluded by extensive autolysis) a necropsy was performed and the tumour examined histologically. We tried to differentiate clinically between papillomas and carcinomas as soon as they developed, by assessing the degree of infiltration by palpation. All tumours registered as carcinomas were histologically verified. Infiltration below the *musculus panniculus* was used as the criterion of malignancy.

*Cell-kinetic studies*.—Sixteen extra animals were used: 4 animals untreated, 4 received 4 applications of MCA, 4 received 4 of TPA, and 4 were exposed to 4 alternating applications, MCA-TPA-MCA-TPA. In all cases, the animals were killed 3 days after the last application and cell-kinetic studies made. The animals were given 0·15 mg Colcemid (Ciba) in 0·5 ml saline i.p. at 08:00, and 3·5 h later (at 11:30) the animals were given 30 $\mu$Ci [$^3$H]dT in 0·5 ml distilled water i.p. The animals were killed at 12:00, 4 h after the Colcemid injection, and 0·5 h after the [$^3$H]dT injection.

The animals were killed by fracture of the neck, and immediately skinned. Small pieces of skin were fixed in formalin and processed for histological examination and autoradiography. These were dehydrated, embedded in paraffin, cut at 5 $\mu$m, and subjected to autoradiography by being dipped in Kodak NTB 2 film emulsion diluted with distilled water 1:1, exposed for 2 weeks, developed and stained with haematoxylin. In these sections, the number of basal cells, the number of non-basal cells, the number of mitotic figures and the number of labelled cells were counted in 40 fields with objective $\times 100$ and eyepiece $\times 12{\cdot}5$, comprising $918 \pm 32$ basal cells in the untreated mouse.

From another part of the skin, epidermal slices were cut with an electrokeratotome (Skjaeggestad, 1964). Single-cell suspensions of basal cells were prepared by trypsin treatment, removal of the differentiating cell layer and shaking (Laerum, 1969; Clausen *et al.*, 1976). The basal cells were fixed in ethanol, treated with RNase and stained with ethidium bromide (Göhde & Dittrich, 1971). The DNA frequency distributions were obtained with an ICP 11 pulse cytophotometer (Phywe AG, Göttingen, W. Germany). Each histogram represented 10,000–20,000 cells. The proportions of cells with $G_1$, S and $G_2+M$ DNA content were calculated by planimetry (Clausen *et al.*, 1976).

The uptake of [$^3$H]dT per $\mu$g epidermal DNA was studied in other pieces of the mouse skin. Epidermis was separated from dermis by a brief heat treatment. Specimens from 2 animals were pooled, homogenized in 5 ml of 0·2N perchloric acid at +4°C and centrifuged (10,000 *g*). The pellet was washed (2 × 2·5 ml) with ice-cold 0·2N perchloric acid and once with 4 ml ethanol/ether (1:1, v/v). DNA in the pellet was hydrolysed in 0·5N perchloric acid at 90°C for 10 min. After centrifugation, duplicate aliquots of hydrolysed DNA (0·3 ml) were determined for radioactivity in a Packard Tri-Carb liquid-scintillation spectrophotometer. The DNA content in the supernatant was determined by the diphenylamine method of Burton (1968).

*Statistical evaluation.*—The results are presented as the tumour *rates* (the percentage of tumour-bearing and cancer-bearing animals in relation to the number of animals alive at appearance of the first papilloma with respect to time) and the tumour *yields* (the cumulative occurrence of all skin tumours and carcinomas with respect to time) in the 4 groups. For the graphs of the yields, the values were first adjusted to equal size of starting group (= 50 mice).

To evaluate differences in tumour rate, we have used the methods for "non-incidental" tumours basically described by Peto (1974) and elaborated with a computer-based test programme by Peto *et al.* (1980). This programme takes care of varying mortality rates (see Table I) among the experimental groups. To evaluate the cumulative tumour-yield curves, we have calculated age-adjusted cumulative tumour-yield curves, adjusting for varying mortality, and then used the method of Gail *et al.* (1980) based on multiple times to tumour, Method 3.

### RESULTS

### *Survival*

Table I shows the survival of the animals in the 4 groups. Group 1 had the lowest death rate. There was no difference between the death rates for Groups 2 and 3, but Group 4 showed the highest death rate at the end of the observation period. However, a log-rank test showed no significant differences between the death rates in the various groups.

### *Cell kinetics*

Table II shows the kinetic data for an untreated, extra control group and for the experimental Groups 2, 3 and 4. The values are those actually observed just before the 5th application in each full treatment schedule. The hyperplasia was most pronounced after 4 TPA applications, but the difference between this hyperplasia and those provoked by 4 applications of MCA or by MCA-TPA-MCA-TPA was not significant. MCA (*not* TPA) led to the highest labelling index, DNA-specific activity and fraction of cells in S. Probably the flux of cells through S was also highest after 4 MCA applications. The mitotic rate was increased after all 3 types of treatment.

TABLE I.—*Percentage survival*

| Treatment group | Months of observation | | | | | | | | | | | | | |
|---|---|---|---|---|---|---|---|---|---|---|---|---|---|---|
| | 0 | 1 | 2 | 3 | 4 | 5 | 6 | 7 | 8 | 9 | 10 | 11 | 12 | 13 |
| 1 | 100 | 100 | 100 | 100 | 98 | 98 | 98 | 98 | 94 | 91 | 90 | 83 | 79 | 64 |
| 2 | 100 | 100 | 100 | 100 | 100 | 98 | 94 | 88 | 76 | 71 | 68 | 62 | 52 | 52 |
| 3 | 100 | 100 | 98 | 98 | 98 | 96 | 85 | 85 | 81 | 77 | 73 | 63 | 56 | 56 |
| 4 | 100 | 99 | 99 | 99 | 99 | 96 | 92 | 91 | 86 | 76 | 70 | 51 | 22 | 22 |

TABLE II.—*Cell kinetic effects 3 days after the last treatment*

| Variable | Untreated controls | MCA x 4 | TPA x 4 | MCA-TPA-MCA-TPA |
|---|---|---|---|---|
| *Cells/40 fields* | | | | |
| Basal cells | 918 ± 32 | 929 ± 24 | 926 ± 18 | 933 ± 12 |
| Supra-basal cells | 611 ± 48 | 856 ± 29 | 1000 ± 74 | 894 ± 21 |
| Average no. of cell layers | 1·67 | 1·92 | 2·08 | 1·96 |
| Relative hyperplasia (increase in no. of supra-basal cells) | 1·00 | 1·37 | 1·61 | 1·43 |
| *Mitotic rate/40 fields/h* | 0·80 ± 0·12 | 2·60 ± 0·50 | 3·30 ± 0·25 | 3·30 ± 0·51 |
| *Labelled cells in 40 fields* | 50 ± 5 | 88 ± 8 | 72 ± 4 | 83 ± 6 |
| *Specific activity* of DNA (ct/min/μg) | 28·0 | 47·5 | 29·5 | 36·2 |
| *Flow-cytometry results* (% of cells in each phase) | | | | |
| $G_1$ | 85 ± 3 | 83 ± 1 | 87 ± 1 | 82 ± 3 |
| S | 11 ± 2 | 13 ± 2 | 6 ± 1 | 10 ± 2 |
| $G_2$ + M | 4 ± 1 | 5 ± 1 | 7 ± 1 | 8 ± 1 |

FIG. 1.—The tumour rate (*i.e.* tumour-bearing animals as % of those alive at the appearance of the first tumour) during the observation period for each of the 4 experimental groups. Group 1 (—··—) got 4 applications of 470 nmol MCA, Group 2 (·····) got 4 applications of MCA, followed by 4 applications of 20 nmol TPA. Group 3 (– – – –) got 4 applications of TPA, followed of 4 of MCA. Group 4 (——) got 4 applications of each of the substances alternately, *i.e.* MCA-TPA-MCA-TPA, etc. Applications were given twice weekly.

FIG. 2.—The carcinoma rate (*i.e.* carcinoma-bearing animals as % of those alive at appearance of the first carcinoma) during the observation period for each of the 4 experimental groups (Symbols as in Fig. 1).

The differences between the kinetic effects of the 3 treatment schedules were small; they all provoked significant hyperplasia with increased rates of cell proliferation. The turnover time of epidermal cells was probably also shortened, because the hyperplasia was less than would have been expected if the maturation time had remained unchanged.

*Tumour rates (papillomas and carcinomas together)*

Fig. 1 shows the tumour rates in the 4 groups. After 10 months all surviving animals in Groups 2, 3 and 4 were tumour-bearing. In Group 1, 65% of the animals had tumours after 13 months. The significant difference between Group 1 and each of the other 3 is obvious. Statistical evaluations of the tumour rates according to the method of Peto *et al.* (1980) for "non-incidental" tumours are shown in Table III. There was no significant difference between Groups 2 and 3. The curve for Group 4 constantly ran higher than the others. The difference between Group 4 and the average of 2 and 3 was very significant.

TABLE III.—*Statistics for tumour rate*

| Group comparison | Obs/Exp | One-tailed $P$ for positive trend | $P$ for heterogeneity $\chi^2$ | Significance |
|---|---|---|---|---|
| All tumours: | | | | |
| 2 } 3 | 1·05 0·95 | 0·72 | 0·57 | NS |
| 2 } 4 | 0·83 1·13 | 0·01 | 0·03 | High |
| 3 } 4 | 0·79 1·15 | 0·005 | 0·01 | Very high |
| 2+3 } 4 | 0·86 1·21 | 0·002 | 0·003 | Very high |
| Carcinomas: | | | | |
| 2 } 3 | 1·05 0·95 | 0·65 | 0·70 | NS |
| 2 } 4 | 0·83 1·12 | 0·10 | 0·20 | NS |
| 3 } 4 | 0·80 1·13 | 0·07 | 0·14 | NS |
| 2+3 } 4 | 0·85 1·20 | 0·04 | 0·08 | Marginal |

*Carcinoma rates*

Fig. 2 shows the carcinoma rates in the 4 groups.

The general trends here were the same as for total tumours. A very significant difference between treatment with MCA alone and any of the combined treatments is obvious. The results for the carcinoma rates were assessed by the statistical method of Peto *et al.* (1980) and are shown in Table III. There was no significant difference between Groups 2 and 3, 2 and 4, or 3 and 4.

However, when the result of Group 4 was compared to Groups 2 and 3 together, the carcinoma rate was just significantly higher for the alternating-treatment schedule.

FIG. 3.—Adjusted tumour yield (*i.e.* total no. tumours adjusted for mortality, see text) during the observation period for each of the 4 experimental groups (Symbols as in Fig. 1.)

*Tumour yields (papillomas and carcinomas together)*

Fig. 3 shows the age-adjusted tumour yields for the 4 groups. The tumour-yield curves revealed generally the same patterns as those for tumour rates. The very significant difference between Group 1 and the other 3 is obvious. The method of Peto *et al.* (1980) is not suitable for total number of tumours. An assessment of the differences between the groups with Gail *et al.* (1980) Model 3 is shown in Table IV. There was no significant difference between Groups 2 and 3. The tumour yield for Group 4 ran higher than the others for the first 8 months, and the difference between Group 4 and Groups 2 and 3 together was significant during this period. Later, however, there was no difference between the groups and the final tumour yields were equal.

*Carcinoma yields*

Fig. 4 shows the age-adjusted carcinoma yields for the 4 groups. The curves

TABLE IV.—*Statistics for tumour yield*

| Group 4 *vs* Groups (2+3) | Final relative odds | $\chi^2$ | *P* |
|---|---|---|---|
| All tumours | | | |
| 1 | 1·62 | 6·98 | <0·001 |
| 2 | 1·58 | 10·84 | <0·001 |
| 3 | 1·46 | 10·32 | <0·001 |
| 4 | 1·48 | 12·85 | <0·001 |
| 5 | 1·38 | 9·97 | <0·01 |
| 6 | 1·37 | 10·75 | <0·01 |
| 7 | 1·27 | 6·76 | <0·01 |
| 8 | 1·21 | 4·76 | <0·05 |
| 9 | 1·15 | 2·59 | <0·20 |
| 10 | 1·10 | 1·25 | <0·30 |
| Summary | | | |
| 29 | 1·00 | 0·00 | 1 |
| Carcinomas | | | |
| 1 | 1·52 | 4·01 | <0·05 |
| 2 | 1·39 | 2·96 | <0·10 |
| 3 | 1·40 | 3·17 | 0·07 |
| 4 | 1·40 | 3·17 | 0·07 |
| Summary | | | |
| 5 | 1·40 | 3·17 | 0·07 |

FIG. 4.—Adjusted carcinoma yield (*i.e.* total no. all carcinomas adjusted for varying mortality, see text) during the observation period for each of the 4 experimental groups (Symbols as in Fig. 1.)

run generally parallel to those for tumour yields. The highly significant difference between the adjusted curve for Group 1 and each of the other 3 is obvious. An assessment with Gail *et al.* model is shown in Table IV. There was no difference between Groups 2 and 3. The curve for Group 4 always ran higher than these, and the difference suggested a higher (though not significant) risk for the alternating treatment schedule.

DISCUSSION

The 2-stage theory of carcinogenesis is basically simple and attractive; it can easily be presented schematically (Boutwell, 1978; Hecker, 1978) and it has all the qualities of a paradigm (Kuhn, 1970).

Repeated applications of a promoter (*e.g.* TPA) strongly potentiates the effect of a single relatively small dose of a complete hydrocarbon carcinogen, and the efficacy of the classical 2-stage protocol in papilloma production is indisputable. The 2-stage *theory*, however, is an interpretation with many corollaries. Its basic assumption is that the synergism is due to an essential, qualitative difference between initiation and promotion (Laerum & Iversen, 1981). One of the most important corollaries of the 2-stage theory is that initiation must come *first*, for maximum tumour yield.

Our results show, however, that at the dose levels used in this study, the inverted treatment schedule was almost as effective as the classical one. Thus, the results of Baba *et al.* (1967) were basically confirmed. It may be objected that both our doses of MCA and those of Baba *et al.* were too high, and represented a completely carcinogenic, not an initiating, dose. This is accepted, but still the results deserve attention.

It was shown already by Mottram (1944) that promoter treatment *before* initiation gave a high tumour incidence, and similar results were demonstrated by Roe (1959). Pound (1963) showed that painting mouse skin with croton oil before an injection of urethane led to an augmented tumour yield in the pretreated area. Tannenbaum *et al.* (1964) showed that pretreatment of mouse skin with croton oil caused a small but significant increase in the incidence of skin tumours and carcinomas, when the mice were thereafter treated with a complete carcinogen. Shinozuka & Ritchie (1967) reported that the yield of papillomas induced by a single application of 7,12-dimethylbenz(a)anthracene (DMBA) followed by repeated applications of croton oil, could be increased by an application of croton oil 23 h *before* the carcinogen, and the same was true when a single injection of urethane was used as initiator.

Pound (1968) showed that treatment with acetic acid or croton oil 24 h before a single application of a complete hydrocarbon carcinogen produced more tumours. Goerttler & Loehrke (1976) showed that treatment of mouse skin with TPA, followed by a small dose of DMBA, and followed again by continued treatment by TPA alone, increased the tumour yield considerably over treatment with a carcinogen plus TPA, but without pretreatment. The orthodox explanation of these results is that the rate of DNA synthesis, or the rate of cellular proliferation, at the time of an application of an electrophilic hydrocarbon carcinogen is of great importance for the final tumour production. Pretreatment with a promoter increases the rate of epidermal proliferation, which increases the binding of the ultimate carcinogen to DNA, and thus one maintains that the two-stage theory, which says that initiation must come *first*, is not invalidated by such results.

Our cell-kinetic results, however, show that at the dose levels used here there is no significant difference between the proliferative stimulation of the epidermis by primary treatment with either MCA or TPA. The rate of cell proliferation is greatly increased by both TPA and MCA treatment, and the two substances provoke comparable hyperplasias. Cell-kinetic alterations produced by TPA cannot therefore explain why the inverted experiment was as effective as the traditional one. It can thus be concluded that the reverse experiment is not innocuous (not even less innocuous), and, under these experimental conditions, initiation *need not* come first.

The reason why the reverse experiment usually ends with few or no tumours may be that in a classical 2-stage experiment the initiating dose of complete carcinogen is applied to a very thin, "virgin", mouse epidermis. What matters for carcinogenesis is probably the effective dose of the ultimate carcinogen reaching some critical macromolecules (often DNA?) in the basal cells. When, in the reverse situation, a small initiating dose of a carcinogen is given topically after weeks of TPA treatment, it is applied to an epidermis that is hyperplastic and has a high rate of cell turnover. The dose reaching the basal cells is probably only a fraction of what would reach these cells in an undisturbed epidermis. The rapid proliferation also reduces the persistence of the carcinogen in the epidermis, and the probability of a transformed cell being shed by the rapid proliferation is probably much greater than in the unstimulated condition. Finally, the observation period after the final delivery of the initiator has always been short.

We therefore feel that our results, together with the reports mentioned, cast some doubt on one of the basic corollaries of the 2-stage theory.

The difference between the tumour production provoked by MCA alone and by the combined treatment shows that TPA and MCA act synergistically and are not only additive. Five paintings with 17 nmol TPA give very few tumours within 13 months (but several later) as shown previously (Iversen & Iversen, 1979).

Thus the synergism between MCA and TPA is considerable and, under the conditions used here, it is manifested whatever the sequence of application.

Our study also indicates a trend for further augmented tumour production following systematic *alternating* treatments with a strong and a weak carcinogen. This is probably the commonest situation in human carcinogenesis, where risk factors in the industrial environment continuously alternate with risk factors related to personal life style. Already Berenblum (1941) showed that alternate applications of promoter and initiator gave many tumours on mouse skin. Salaman & Roe (1953) got the highest tumour yield from alternate treatments of urethane and croton oil over a period of 21 weeks. Schoental (1963) demonstrated a synergistic effect of hydrocarbon and nitrosourethane carcinogens given alternately. Boutwell (1976) reported the results of weekly paintings with different doses of DMBA alternating with a constant weekly dose of croton oil (0·5% solution) and compared these results with those after repeated weekly treatments with carcinogen alone. The results were that, for each dose level of the strong carcinogen, there was a higher tumour yield when croton oil was also given in sequential paintings. The increase in tumorigenicity after sequential delivery of carcinogens may also be relevant to occupational carcinogenesis. Many workers are also cigarette smokers, and they are therefore exposed to an industrial carcinogen in their working time and to cigarette smoke in their leisure time (see *e.g.* Kreyberg, 1978). Meurman *et al.* (1974) estimated the risk factor of combined, alternating exposure in the following way (using a multiplicative model and certain specific assumptions):

| | |
|---|---|
| non-smokers and non-asbestos workers | 1·0 |
| asbestos workers (non-smokers) | 1·4 |
| smokers (non-asbestos workers) | 12·0 |
| smokers and asbestos workers combined | 17·0 |

Selikoff *et al.* (1968) estimated the combined risk factor to be as high as 92. Our results indicate that the highest risk of tumour development in skin carcinogenesis also occurs after *alternate* exposures to strong and weak carcinogens.

We are grateful to Dr J. Wahrendorf at the IARC in Lyon, France; and to Dr M. H. Gail, NCI, Bethesda, U.S.A., for giving us their computer-based statistical models. We also thank the technical staff and the secretariat at the Institute of Pathology, University of Oslo, for excellent assistance.

### REFERENCES

BABA, T., AOKI, K. & ISHII, M. (1967) Relation between the so-called two-phase theory and summation of carcinogenesis. *Gann*, **58**, 161.

BERENBLUM, I. (1941) The co-carcinogenic action of croton resin. *Cancer Res.*, **1**, 44.

BERENBLUM, I. & HARAN, N. (1955). The significance of the sequence of initiating and promoting actions in the process of skin carcinogenesis in the mouse. *Br. J. Cancer*, **9**, 268.

BOUTWELL, R. K. (1964) Some biological aspects of skin carcinogenesis. *Progr. Exp. Tumor Res.*, **4**, 207.

BOUTWELL, R. K. (1976) The biochemistry of preneoplasia in mouse skin. *Cancer Res.*, **36**, 2631.

BOUTWELL, R. K. (1978) Biochemical mechanism of promotion. In *Carcinogenesis*, Vol. 2 (Eds. Slaga *et al.*). New York: Raven Press. p. 49.

BURTON, K. (1968) Determination of DNA concentration with diphenylamine. *Methods Enzymol.*, **12**, 163.

CLAUSEN, O. P. F., LINDMO, T., SANDNES, K. & THORUD, E. (1976) Separation of mouse epidermal basal and differentiating cells for microflow fluorometric measurements. *Virch. Arch. B. Cell Pathol.*, **20**, 261.

GAIL, M. H., SANTNER, T. J. & BROWN, C. C. (1980) An analysis of comparative carcinogenesis experiments based on multiple times to tumor. *Biometrics*, **36**, 255.

GOERTTLER, K. & LOERHKE, H. (1976) Improved tumour yields by means of a TPA-DMBA-TPA variation of the Berenblum-Mottram experiment on the back skin of NMR1 mice. *Exp. Pathol.*, **12**, 336.

GÖHDE, W. & DITTRICH, W. (1971) Impulsfluorometrie,—ein neuartiges Durchflussverfahren zur ultraschnellen Mengenbestimmung von Zellinhaltstoffen. *Acta Histochem.* **10**, (Suppl.) 429.

HECKER, E. (1978) Structure-activity relationship in diterpene esters irritant and cocarcinogenic to mouse skin. In *Carcinogenesis*, Vol. 2 (Eds. Slaga *et al.*). New York: Raven Press. p. 11.

IVERSEN, U. M. & IVERSEN, O. H. (1979). The carcinogenic effect of TPA (12-O-tetradecanoylphorbol-13-acetate) when applied to the skin of hairless mice. *Virch. Arch. B. Cell Pathol.*, **30**, 33.

KREYBERG, L. (1978) Lung cancer in workers in a nickel refinery. *Br. J. Indust. Med.*, **35**, 109.

KUHN, T. S. (1970) The structure of scientific revolutions. In *Int. Encyclopedia Unified Science*, Vol. 2 (2nd Ed.), Chicago: University Press. p. 172.

LAERUM, O. D. (1969) Oxygen consumption of basal and differentiating cells from hairless mouse epidermis. *J. Invest. Dermatol.*, **52**, 204.

LAERUM, O. D. (1973) Reticulum cell neoplasms in normal and benzene treated hairless mice. *Acta Pathol. Microbiol. Scand., Sect. A*, **81**, 57.

LAERUM, O. D. & IVERSEN, O. H. (eds.) (1981). The biology of skin cancer, excluding melanomas. *UICC Technical Report Series 60, Report No. 13.* (Chap. 6, Discussion) Geneva: UICC.

MEURMAN, L. O., KIVILUOTO, R. & HAKAMA, M. (1974) Mortality and morbidity among the working population of anthophyllite asbestos miners in Finland. *Br. J. Indust. Med.*, **31**, 105.

MOTTRAM, J. C. (1944) A developing factor in experimental blastogenesis. *J. Pathol. Bacteriol.*, **54**, 181.

PETO, R. (1974). Guidelines on the analysis of tumour rates and death rates in experimental animals. *Br. J. Cancer*, **29**, 101.

PETO, R., PIKE, M. C., DAY, N. E. & 6 others (1980). Guidelines for simple, sensitive significance tests for carcinogenic effects in long-term animal experiments. In *Long-term and Short-term Screen-*

*ing Assays for Carcinogens: A Critical Appraisal.* IARC Monogr., Suppl. 2, (Eds. Montesano *et al.*). p. 311.

POUND, A. W. (1963) The localization of the influence of croton oil stimulation on tumour initiation by urethane in mice. *Austral. J. Exp. Biol. Med. Sci.*, **41,** 73.

POUND, A. W. (1968) Carcinogenesis and cell proliferation. *N.Z. Med. J.*, **67,** 88.

ROE, F. J. C. (1959) The effect of applying croton oil *before* a single application of 9,10-dimethyl-1,2-benzanthracene (DMBA). *Br. J. Cancer*, **13,** 87.

SALAMAN, M. H. & ROE, F. J. C. (1953) Incomplete carcinogens: Ethyl carbamate (urethane) as an initiator of skin tumour formation in the mouse. *Br. J. Cancer*, **7,** 472.

SELIKOFF, I. J., HAMMOND, E. G. & CHURG, J. (1968) Asbestos exposure, smoking and neoplasia. *JAMA*, **204,** 106.

SHINOZUKA, H. & RITCHIE, A. C. (1967) Pretreatment with croton oil, DNA synthesis, and carcinogenesis by carcinogen followed by croton oil. *Int. J. Cancer*, **2,** 77.

SHOENTAL, R. (1963) Experimental induction of squamous carcinoma of the lung, oesophagus and stomach: The mode of their induction. *Acta Unio Int. Contra Cancrum*, **19,** 680.

SKJAEGGESTAD, Ø. (1964) Experimental epidermal hyperplasia in mice. Relation to carcinogenesis. *Acta Pathol. Microbiol. Scand.* (*Suppl.*) **169,** 1.

TANNENBAUM, A., VESSELINOVITCH, S. D. & SILVERSTONE, H. (1964) Increased induction of skin tumors by pretreatment with croton oil. *Cancer Res.*, **24,** 361.

WEINSTEIN, I. B. (1978). Current concepts on the mechanism of chemical carcinogenesis. *Cancer Bull.*, **29,** 144.